# Visualization of spatial and temporal temperature distributions with magnetic particle imaging for liver tumor ablation therapy

**DOI:** 10.1038/s41598-020-64280-1

**Published:** 2020-05-04

**Authors:** J. Salamon, J. Dieckhoff, M. G. Kaul, C. Jung, G. Adam, M. Möddel, T. Knopp, S. Draack, F. Ludwig, H. Ittrich

**Affiliations:** 1 Department for Diagnostic and Interventional Radiology and Nuclear Medicine, University Medical Center Hamburg-Eppendorf, 20246 Hamburg, Germany; 2 Section for Biomedical Imaging, University Medical Center Hamburg-Eppendorf, 20246 Hamburg, Germany; 3 Institute for Biomedical Imaging, Hamburg University of Technology, 21073 Hamburg, Germany; 4 Institute of Electrical Measurement Science and Fundamental Electrical Engineering, TU Braunschweig, 38106 Braunschweig, Germany

**Keywords:** Targeted therapies, Experimental models of disease, Translational research, Imaging techniques

## Abstract

Temperature-resolved magnetic particle imaging (MPI) represents a promising tool for medical imaging applications. In this study an approach based on a single calibration measurement was applied for highlighting the potential of MPI for monitoring of temperatures during thermal ablation of liver tumors. For this purpose, liver tissue and liver tumor phantoms embedding different superparamagnetic iron oxide nanoparticles (SPION) were prepared, locally heated up to 70 °C and recorded with MPI. Optimal temperature MPI SPIONs and a corresponding linear model for temperature calculation were determined. The temporal and spatial temperature distributions were compared with infrared (IR) camera results yielding quantitative agreements with a mean absolute deviation of 1 °C despite mismatches in boundary areas.

## Introduction

Minimal invasive treatment of hepatic tumors based on thermal ablation has become an important alternative to traditionally applied therapies, e.g. chemo- or radiation therapy or surgical resection, especially if surgery is associated with higher risk of complications^1^. Guidance of the thermal ablation process is commonly performed with ultrasound (US), fluoroscopy, computed tomography (CT) or magnetic resonance imaging (MRI)^2^. Whereas US, fluoroscopy and CT provide fast localization of liver tumor and guidance of ablation devices with adequate image contrast, a real-time measurement of the tissue temperatures induced by the thermal ablation processes is clinically only facilitated by MRI. Such temperature controlled interventional guidance enables a direct control of the induced tissue temperature distribution by the operator and consequently improves the safety and success of the therapy^3,4^. However, the availability of open MRI systems – required for these interventional applications – is quite limited. Higher financial investments are required and a combination of high spatial and temporal resolution is hampered by lower field strength (1T-1.5 T) compared to closed scanners (>3.0 T). Therefore, alternative fast and temperature-resolved imaging techniques are of high interest – such as magnetic particle imaging (MPI) which is characterized by frame rates of up to 46 frames/s. In principle, MPI scanners can be designed as open bore or even floating table systems^5^ allowing proper access to the investigated object – in the distant future possibly a patient. One promising concept proposed a setup for an open bore FFL MPI scanner without the need for mechanical rotation, where a comparable large field of view (FOV) can be scanned in a sufficient time span, which is needed for clinical usage^6^.

Magnetic particle imaging (MPI) was initially presented to measure *in vivo* distributions of superparamagnetic iron oxide nanoparticles (SPION) in cardiovascular applications with high temporal and spatial resolution^7^. MPI has rapidly developed and several other possible clinical and preclinical applications were presented, for instance: It was shown that MPI allows for preclinical stem cell tracking^8,9^. In the field of Neuroimaging MPI proved to be able to assess the hemodynamics of intracranial aneurysms^10^ and detect ischemic stroke in a mouse model with high sensitivity and high temporal resolution^11^. It has been shown suitable to detect small gut bleedings^12^ and to image pulmonary perfusion in small animal models^13^. MPI might be a useful tool for guiding catheter interventions by multicolored imaging^14^ and steering of interventional instruments^15^. Since the used particles are finally taken up by different organs, especially the mononuclear phagocytic system of the liver, MPI enables a direct visualization of healthy liver tissue^16,17^. The missing accumulation of these SPION in primary liver tumors^18^, e.g. hepato- or cholangiocellular carcinoma (HCC, CCA) or metastases, results in an indirect tumor imaging. In particular, tumors are causing signal gaps or drops in the measured particle distribution depending on the tumor structure and spatial MPI resolution. For instance, a heterogeneous tumor in the range of the spatial resolution could be overseen with MPI due to the partial particle uptake. Therefore, a second modality, e.g. CT or MRI either separately to the MPI or in a hybrid system^19,20^, must be used as an anatomical reference for reliable tumor detection^21^.

The magnetization dynamics of SPION applied in MPI depend significantly on temperature, which can be utilized for precise temperature measurements^22^. In first *in vitro* experiments, it was demonstrated that MPI allows simultaneous imaging of both, particle concentration and particle temperature distribution. For data reconstruction the multi-color approach was utilized and a temperature measurement precision of ± 0.5 °C was estimated^23^. Furthermore, MPI was evaluated for predicting the therapeutic effect of magnetic hyperthermia utilizing iron oxide nanoparticles. In *in vivo* experiments a strong correlation was found between the magnetically induced temperature rise and the average MPI value of the particle “hot spot”^24^.

In this study, we investigate the principle capability of MPI to monitor spatial and temporal temperature distributions in thermal liver tumor ablation therapies. The main challenge of thermal tumor ablation represents the tradeoff between sufficient tumor heating – destroying cancer cells reliably – and tolerable heating of healthy adjacent tissue – ensuring liver functionality. In this context, MPI can be applied to image the temperature distribution of healthy liver tissue surrounding tumors. Thus, MPI will provide direct temperature feedback to ensure adequate heating safety margins. In the case of thermal HCC ablation, tissue heating of at least 50–55 °C for 4–6 minutes – causing irreversible cellular damage – is recommended including a 0.5–1 cm thick safety margin around the HCC^2^. Herewith, an adequate destruction of microscopic invasions inside the healthy tissue is ensured. During thermal ablation, changes of the measured MPI particle distribution can be directly attributed to temperature changes, since the hepatic SPION distribution can be considered quasi-constant after the intravenous SPION injection and hepatic uptake process. Thus, MPI data reconstruction could be performed with one calibration measurement (system matrix) minimizing the calibration effort, e.g., required for multi-color temperature MPI^25^. In order to evaluate the temperature imaging capabilities of MPI in general and of this application in particular, MPI results of locally heated liver and liver tumor phantoms are compared with optical temperature (OT) sensor and infrared (IR) camera measurements.

## Materials and Methods

### Liver tumor ablation phantoms

**Figure 1:**
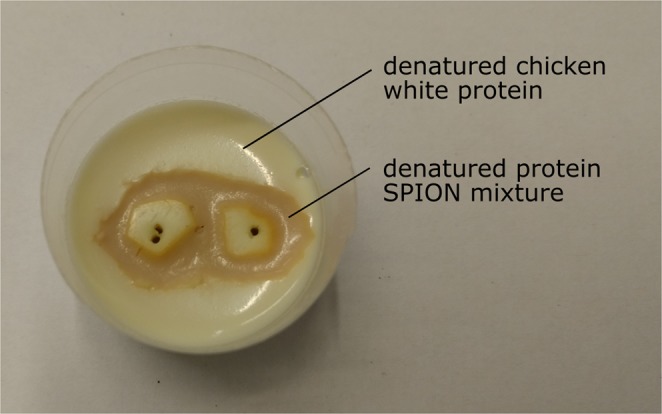
Phantom resembling liver tissue with two tumors after iron oxide contrast agent administration. Healthy liver tissue is represented by denatured chicken white protein with embedded SPION (brown structure) in contrast to SPION-free liver tumors (white spots) and surrounding tissue (white surrounding matrix). The tumors have a diameter of approximately 5 mm, the petri dish has a diameter of 60 mm. The points centrally in the liver tumors are caused by wire placement.

The liver phantoms consist of MPI-suitable SPION embedded in denatured protein. For this purpose, the SPION were gently mixed with dissolved dried egg powder purchased from Sigma Aldrich (St. Louis, MO, USA) and heated in a water bath (80 °C) for 15 minutes. These phantoms represent healthy liver tissue after the specific uptake of SPION with respect to the particle aggregation and mobility status^16^. Corresponding phantoms representing tumorous liver tissue (see Fig. 1) were prepared from SPION-free and SPION-loaded denatured protein. Here, SPION-free areas simulate the missing accumulation of contrast agents in tumors.

Three different iron oxide multi-core nanoparticles were tested for their temperature imaging capabilities, whose composition benefits a quick and efficient liver SPION loading. Ferucarbotran (Resovist, Bayer Pharma AG, Berlin, Germany) was specifically developed for liver MRI investigations and is consequently characterized by rapid clearance from blood stream and predominant liver macrophage uptake^26^. It was successfully utilized in *in vivo* liver MPI experiments; however, image resolution appears to be suboptimal comparing ferucarbotran with MPI optimized SPION. Perimag nanoparticles purchased from micromod Partikeltechnologie GmbH (Rostock, Germany) are stabilized with dextran and exhibit hydrodynamic diameters of around 130 nm. They were optimized for MPI resulting in superior signal spectra^27^ enabling various biomedical imaging applications, e.g. cell tracking^17^. L93 represents a batch of MPI-optimized nanoparticles synthesized by Gunnar Schuetz from Bayer Pharma AG (Berlin, Germany) with dextran stabilization and average hydrodynamic diameter of 66 nm.

### Temperature-dependent MPS

The temperature dependence of the MPI signal spectra of the different SPION embedded in denatured protein were evaluated with a temperature controllable magnetic particle spectrometer (MPS)^28^. The setup comprises a one-dimensional alternating magnetic field excitation with a field amplitude and frequency of 12 mT and 25 kHz, respectively, resembling the performed MPI scans. The simultaneously recorded signal spectra were analyzed for sample temperatures ranging from 20 °C to 80 °C.

### Temperature MPI and reference

Temperature MPI was first evaluated with 100 µL samples containing the different SPIONs embedded in denatured protein. After identifying the most suitable SPION for temperature imaging, especially with respect to temperature sensitivity, (non-)tumorous liver phantoms containing the optimal SPION were prepared and investigated. The scans were performed with a preclinical MPI system (Philips Medical Systems DMC GmbH, Hamburg, Germany/Bruker BioSpin GmbH, Ettlingen Germany). It utilizes the field free point (FFP) approach scanning the field of view along 3D Lissajous trajectories. To guarantee stable system operation with respect to signal disturbances and cover an adequate FoV in the size range of a murine liver lobe while achieving an adequate spatial resolution and good signal-to-noise ratio (SNR), drive field amplitude and gradient strength were set to 12 mT and 1 Tm^−1^ (x-, y-direction)/2 Tm^−1^ (z-direction), respectively. Whereas the resulting FoV with a size of 24 × 24 × 12 mm³ for x-, y- and z-direction can be directly calculated, the spatial resolution and SNR strongly depend on the applied iron oxide nanoparticle system.

As temperature reference, the fiber optic temperature sensor TS5 (Optocon AG, Dresden, Germany) with temperature resolution of 0.1 °C and absolute accuracy of ±1 °C was utilized. In addition, the infrared camera Testo 885 (Testo SE & Co. KGaA, Lenzkirch, Germany) with temperature resolution of 0.1 °C and absolute accuracy of ±2 °C was used as reference for temperature images. With the corresponding 11° × 9° IR telephoto lens an image resolution of approximately 0.5 mm could be realized for the average object-to-camera distance of 0.75 m. For measurements, the camera was installed directly in front of the scanner’s bore opening, thus, imaging phantom surface temperatures in the y-z-plane. IR measurements were started prior to MPI measurements, time between start of IR and MPI data acquisition was measured and IR images recorded prior to the MPI data acquisition were excluded. Image registration between both modalities was performed manually assisted by phantom geometries visible in the IR images. Especially, the apparent temperature differences between the phantom and the surrounding environment (scanner bore) as well as the known phantom geometries enabled a reliable registration.

Phantom heating was achieved by sticking a straight copper wire in the phantom and keeping it inside the phantom during the MPI scan. The copper wire (diameter: 1 mm; length: 80 mm) was heated by eddy currents induced by the magnetic drive field^29^. Thus, phantom heating was directly linked to the MPI image acquisition process. The maximum temperatures in close vicinity (<3 mm) to the copper wire measured with reference sensor TS5 amounted to more than 80 °C. These temperatures represented a thermal equilibrium between heat generation by eddy currents and phantom cooling by system ventilation. In the MPI images no eddy current related artifacts were observed. Whereas the eddy current heating effect was beneficial for this study, it represents a serious safety issue in other MPI applications, e.g. in cardiovascular imaging of stents^29^.

The temperature MPI procedure consisted of the following steps: 1. The phantom with the copper wire was placed in the scanner’s bore according to the center of the FoV. 2. The corresponding temperature reference was positioned and a settling of the phantom temperature to the ambient temperature in the bore of (19 ± 1)°C was ensured to guarantee the same start conditions. 3. The MPI scan was started resulting in simultaneous sample heating and image acquisition. The latter was delayed (typically 25 s) due to the drive field startup process. The MPI scan time was set to reach thermal equilibrium – characterized by a temperature rise smaller than 0.5 °C/min.

MPI data reconstruction was adopted from previous investigations dealing with liver MPI^16,21^ utilizing a calibration measurement ***S*** (system matrix) linking the distribution of the particle iron concentration ***c*** and the measurement vector ***û***:

1 $$Sc=\mathop{u}\limits^{\frown {}}.$$

In practice, a self-developed software framework^30^ programmed in the high-level general-purpose dynamic programming language Julia^31^ utilizing Kaczmarz’s algorithm and Tikhonov regularization^32^ was applied. The basic reconstruction procedure is explained in more detail by Knopp *et al*.^33^. Reconstruction parameters were chosen with respect to image signal-to-noise ratio: Regularization factor *λ* = 1 ∙ 10^–3^, number of iterations *I* = 6, signal-to-noise threshold *SNR* = 3 and number of averaged frames *N* = 500. The reconstruction of a MPI scan was performed with one system matrix measured on a cubic sample (3 × 3 × 3 mm³) filled with the corresponding SPION immobilized in denatured chicken white protein with a particle iron concentration of 100, 51.1 and 42.5 mM for ferucarbotran (Resovist), perimag and L93, respectively. The sample temperature was given by the average ambient temperature in the scanner’s bore.

MPI temperature images ***T***_**MPI,t**_ reflecting the spatial and temporal temperature distribution in SPION-loaded denatured protein – independent of the particle concentration – were calculated as follows:

2 $${{\boldsymbol{T}}}_{{\bf{M}}{\bf{P}}{\bf{I}},{\bf{t}}}=({\boldsymbol{MP}}{{\boldsymbol{I}}}_{{\bf{r}}{\bf{e}}{\bf{l}},{\bf{t}}}-1)\cdot {m}^{-1}+{{\boldsymbol{T}}}_{{\bf{M}}{\bf{P}}{\bf{I}},0}.$$

here, ***MPI***_**rel,t**_ represents the element-wise division of the MPI particle concentration **c** of time frame *t* by the initial particle concentration ***c***_**0**_ – representing the first time frame of the image acquisition process. The element-wise division was performed for each element of ***c***_**0**_ with a value larger than 0.1∙*c*(Fe) of the prepared phantom to ensure reliable signal analysis. Otherwise the element of ***MPI***_**rel,t**_ was excluded. In the following sections, the elements of ***MPI***_**rel,t**_ are referred to as relative MPI signal. Since the real particle concentrations in the phantoms were constant, changes of the elements of ***MPI***_**rel,t**_ could be directly linked to temperature changes via one single particle-dependent temperature coefficient *m*. In practice, *m* was based on temperature MPI scans of the chosen SPION immobilized in denatured protein and corresponding linear regressions (see results section: *Temperature coefficient analysis*). In order to calculate absolute temperature images the initial temperature distribution ***T***_**MPI,0**_ was taken from IR camera reference measurements.

## Results

### Temperature-dependent MPS

**Figure 2:**
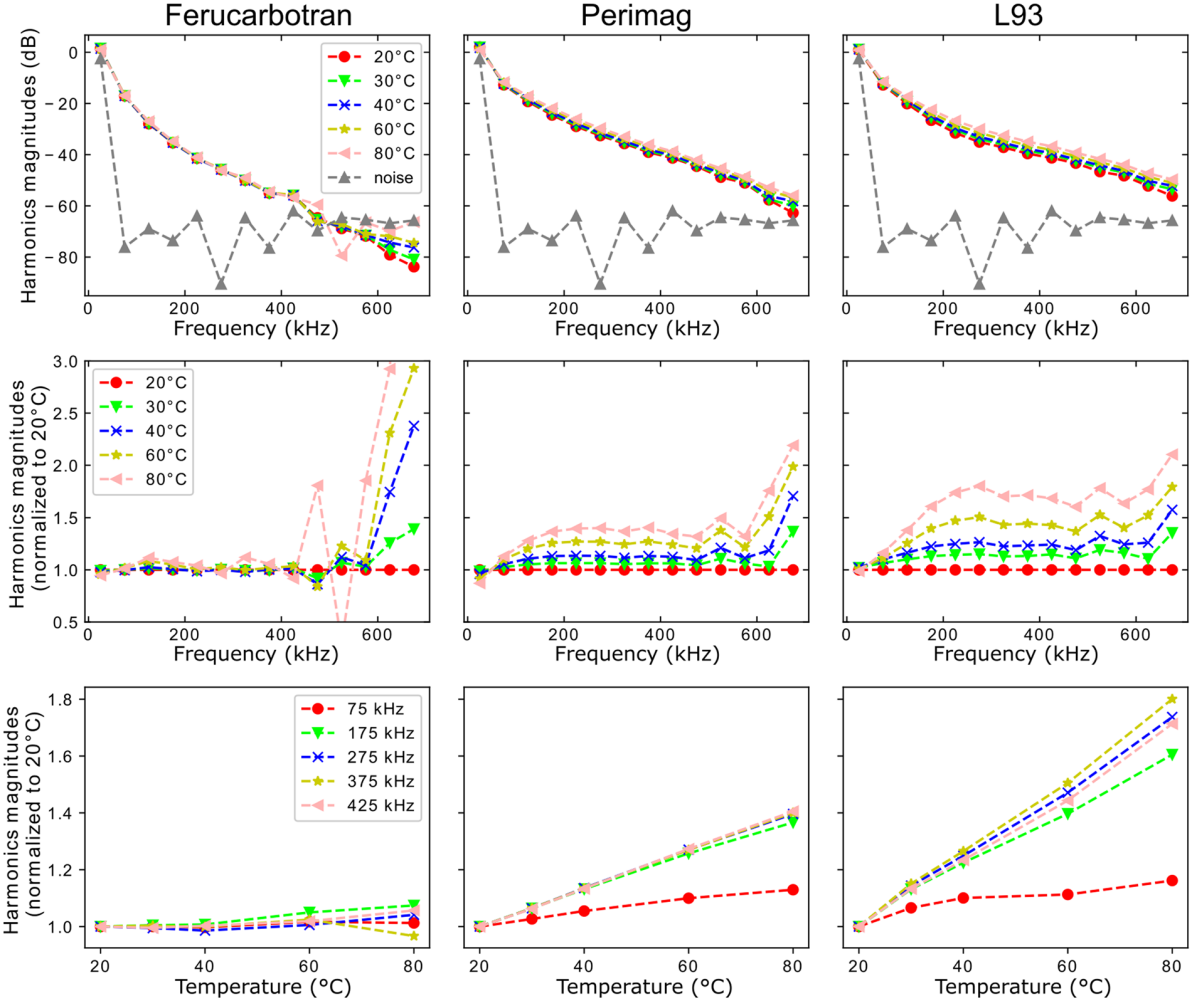
Odd harmonics magnitudes of temperature-dependent MPS results as function of frequency (first row). A normalization of the harmonics magnitudes to the corresponding spectrum recorded at 20 °C (second row) illustrates the magnitudes’ temperature dependence (third row). The dashed lines are guides to the eyes and do not represent possible signal components between the measurement points.

Samples with SPION embedded in denatured protein and volumes of 150 μL were prepared for the temperature-dependent MPS measurements. The particle iron concentrations *c*(Fe) amounted to 50 mM for perimag and L93 as well as 100 mM for ferucarbotran in order to gain a sufficient signal strength. The measurement results, shown in Fig. 2, indicated different temperature MPI performances between the investigated SPION. In detail, the particles’ signal strengths differed – represented by the decay of the harmonics magnitudes. For instance, the harmonics magnitudes of ferucarbotran were characterized by the fastest decay. They dropped below the noise level at around 400 kHz limiting the signal analysis at higher frequencies. In contrast, the harmonics magnitudes of perimag and L93 were usable up to 600 kHz, although the particle iron concentration was only half of the concentration of ferucarbotran. Furthermore, different temperature-dependencies of the particles’ harmonics magnitudes could be observed. The dependence was strongest for L93 followed by perimag. For ferucarbotran, differences between the curves recorded at varying temperatures were hardly visible. Thus, the measured harmonics were normalized to the corresponding harmonics curve recorded at 20 °C. The illustration of these results as a function of frequency (second row) and temperature (third row) clarified the previously described temperature dependence. For perimag and L93, a nearly linear relation between magnitude and temperature was found, which could be approximated as constant between 200 kHz and 600 kHz. This was a prerequisite for a concentration-independent calculation of MPI temperature distributions according to Eq. (2).

### Temperature dependence of MPI signal

**Figure 3:**
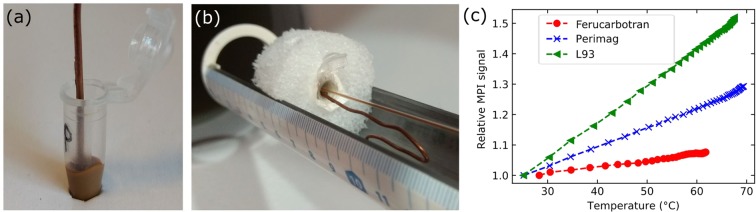
Experimental determination of the SPIONs MPI signal temperature dependencies. (**a**) A copper wire placed for heating purpose in a 100 µL denatured protein sample with embedded perimag. (**b**) Positioning of the sample together with the temperature reference TS5 in the MPI scanner’s bore. (**c**) The relative MPI signal as a function of temperature illustrates an approximately linear relationship to temperature of ferucarbotran, perimag and L93 as described in Eq. (2).

In Fig. 3a, a sample containing 100 µL denatured protein with embedded perimag particles (*c*(Fe) = 51.1 mM) and stuck copper wire is shown. Two similar samples containing ferucarbotran and L93 were prepared additionally. The samples were positioned in the center of the FoV together with temperature reference TS5 according to Fig. 3b. The results of the MPI temperature scans were reconstructed excluding signal components below 80 kHz due to filter instabilities of the MPI scanner. For each SPION, Fig. 3c illustrates the temperature dependence of the relative MPI signal, namely the element of ***MPI***_**rel,t**_ with the highest value in ***c***_**0**_ as a function of temperature measured with reference TS5. These results support the temperature-dependent MPS measurements: an approximately linear relationship between signal and temperature was found, which was highest for L93 followed by perimag and ferucarbotran.

### Temperature coefficient analysis

Comparing the three SPION types, the first experimental results indicated that L93 was most suitable for temperature MPI in terms of harmonics magnitudes and signal temperature dependence. Perimag and ferucarbotran possessed reduced temperature dependencies. In the case of ferucarbotran, the dependence even changed over the whole investigated frequency range. Thus, the following temperature MPI measurements were performed with phantoms based on L93.

**Figure 4:**
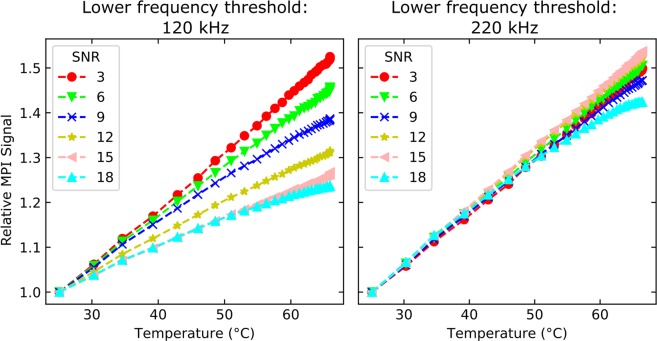
Illustration of the influence of the SNR reconstruction parameter on MPI signal temperature dependence of L93. The L93 measurement data is taken from Fig. 3. Frequencies below 120 kHz (left image) and 220 kHz (right image) were excluded from the underlying reconstructions.

To calculate ***T***_**MPI,t**_, the SPION-specific temperature coefficient *m* had to be determined. In principle, it was directly given by the linear slope of the corresponding curve in Fig. 3. It was assumed that *m* is independent of particle iron concentration if scan parameters and particle immobilization are unchanged. To verify that *m* and, consequently, ***T***_**MPI,t**_ are independent, the L93 dataset from Fig. 3 was reconstructed for different *SNR* thresholds ranging from 3 to 18. The increase of this reconstruction parameter reduced the frequency components considered for reconstruction due to the decay of the frequency components’ magnitudes with frequency and, thus, reflected the dependence of *m* on the given particle iron concentration. In comparison: A reduced particle iron concentration would also result in a reduced number of frequency components considered for reconstruction. Figure 4 demonstrates, that the temperature dependence of the relative MPI signal noticeably varied with *SNR* if signal frequency components below 220 kHz were considered for reconstruction. In contrast, comparable dependencies for varying *SNR* were found if these frequency components were not utilized in the reconstruction by setting the lower frequency threshold to 220 kHz. Only for *SNR* = 18, a clearly different non-linear behavior was observed. In this case, only seven frequency components were considered for reconstruction representing intolerable signal strength for temperature MPI.

For determination of an average *m*, the MPI scan with the L93 sample was carried out three times. Each time five reconstructions were computed with a lower frequency threshold of 220 kHz and a *SNR* threshold of 3, 6, 9, 12 or 15. Linear regressions were performed for each single reconstruction result yielding an average *m* of 0.01186 K^−1^ with a standard deviation of 0.00062 K^−1^. The root mean square of the deviation between these results and the linear model is 0.00294 K^−1^ representing an acceptable uncertainty for temperature calculations.

**Figure 5:**
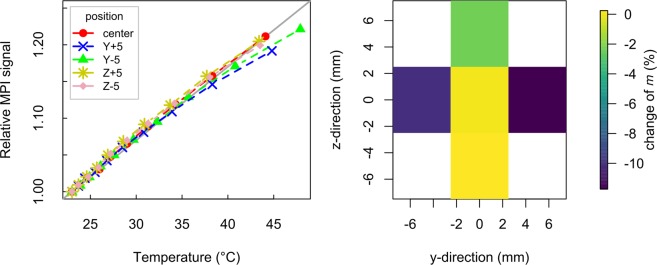
MPI signal temperature dependence of L93 sample shifted 5 mm in positive and negative y- and z-direction in comparison to linear regression of measurement at central position (gray line). Image on the right side represents percentaged change of *m* for different sample positions in comparison to central position.

In order to identify possible spatial dependences of *m* comparable measurements were carried out with the heated L93 sample positioned 5 mm away from the FoV center in the positive and negative y- and z-direction. These directions represent the image plane of the following MPI images, which can be monitored by the reference IR camera. The temperature induced MPI signal changes of all positions show reasonable linear dependencies, see Fig. 5 (left image). Comparing all results with the linear model (average *m* determined at the central position) a root mean square deviation of 0.0051 K^−^1 can be found. Performing linear fits on the dataset of each position, a maximal reduction by 10% (y-direction) and 3% (z-direction) is identified in comparison to *m* determined at the central position, see Fig. 5 (right image). This is still in an acceptable range for the current investigation.

### MPI temperature images

**Figure 6:**
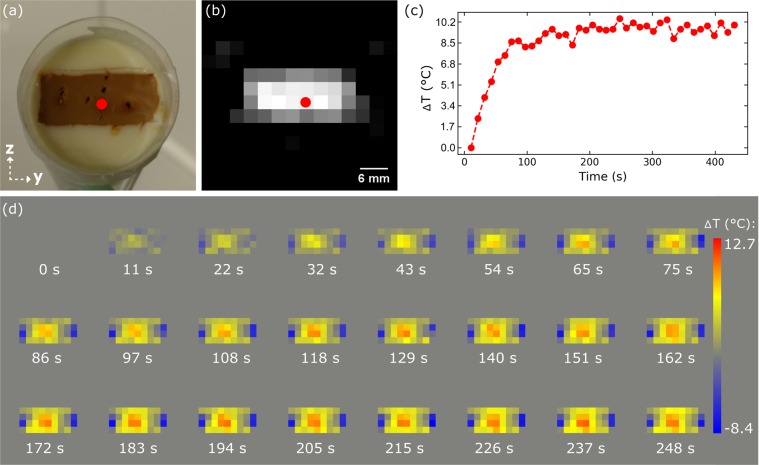
Effect of L93 phantom heating on temporal and spatial changes in MPI temperature images. (**a**) Photography shows phantom and MPI scanner coordinate system. The dark spots on the phantom correspond to locations at which the copper wire was placed. (**b**) Corresponding layer of the initial time point of the reconstructed MPI time series reflects cuboid phantom geometry. Red dot indicates position of copper wire. (**c**) Temperature change at the location of the copper wire obtained from the MPI time series. (**d**) Collection of temperature change maps obtained from the MPI time series at different points of time.

In order to analyze MPI temperature images, a cuboid phantom was prepared (see Fig. 6a). The particle iron concentration of embedded L93 SPION amounts to 12.8 mM. The cuboid side lengths of approximately 22 × 10 × 5 mm^3^ were chosen to guarantee a full coverage of the phantom by the FoV. The heating copper wire was centrally stuck into the phantom (red dot). The corresponding layer of the MPI image in Fig. 6b – representing the absolute particle concentration ***c***_**0**_ – clearly reflects the phantoms cuboid geometry.

The MPI temperature change of the element marked with a red dot is plotted as a function of time in Fig. 6c. Here, a fast temperature rise of more than 8 °C is found within the first 100 s. After more than 200 s, the signal stabilizes 10 °C above the start temperature due to thermal equilibrium between heat generation and cooling. The change of the spatial MPI temperature distribution is shown in Fig. 6d for different time points. In detail, the appearance and expansion of a central temperature hot spot inside the phantom can be observed. However, also negative temperature changes appear at the phantom’s edges in y-direction.

**Figure 7:**
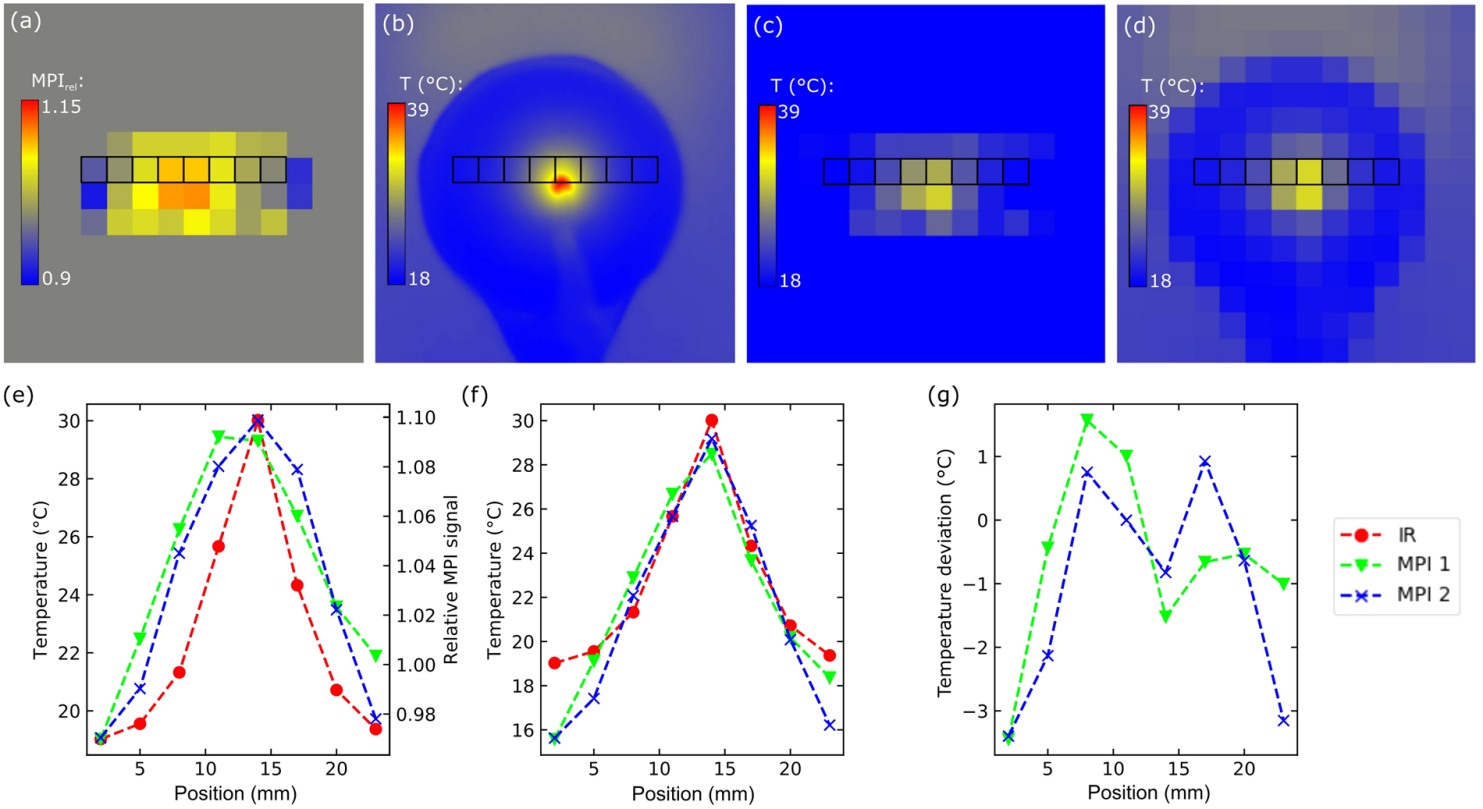
Comparison of MPI and IR temperature images of centrically heated L93 phantom at *t* = 151 s: (**a**) Relative MPI signal distribution, (**b**) original IR image, (**c**) MPI temperature image calculated according to Eq. (2) and (**d)** IR temperature image with resolution downsized to MPI resolution. (**e**) The rectangle-marked elements of the pixelated IR image are compared with corresponding elements of two different layers (MPI 1 and 2) of the relative MPI signal distribution and (**f**) MPI temperature image. (**g**) The resulting temperature deviations between IR camera and MPI temperature image are presented.

In Fig. 7a comparison of temperature MPI and IR camera results recorded on the L93 phantom from Fig. 6a is presented. In addition to images, line profiles are shown, which are marked in the images with black rectangles. The resolution of the original IR camera image was downsized to the MPI resolution to enable a representative image and line profile comparison. Whereas the relative MPI signal, as image and profile, showed a fair qualitative agreement with IR camera results, the MPI temperature image and the corresponding profiles yield good quantitative agreements. MPI data from two layers are presented for comparison. The top layer (MPI 1) is shown in the images. The bottom layer (MPI 2) represents the slice of the reconstructed MPI image stack directly below the top layer, thus, not visible for the IR camera. The resulting temperature deviations between IR camera and ***T***_**MPI,t**_ (line profiles) are illustrated in Fig. 7g with a maximum deviation of 3 °C at the phantom’s edges. ***T***_**MPI,0**_ required for the calculation of ***T***_**MPI,t**_ was based on the IR camera image taken at the time of the first MPI frame. Since MPI data acquisition was delayed with respect to drive field start up (around 25 s), ***T***_**MPI,0**_ does not represent the starting point of the heating process, which is characterized by a homogenous temperature distribution.

**Figure 8:**
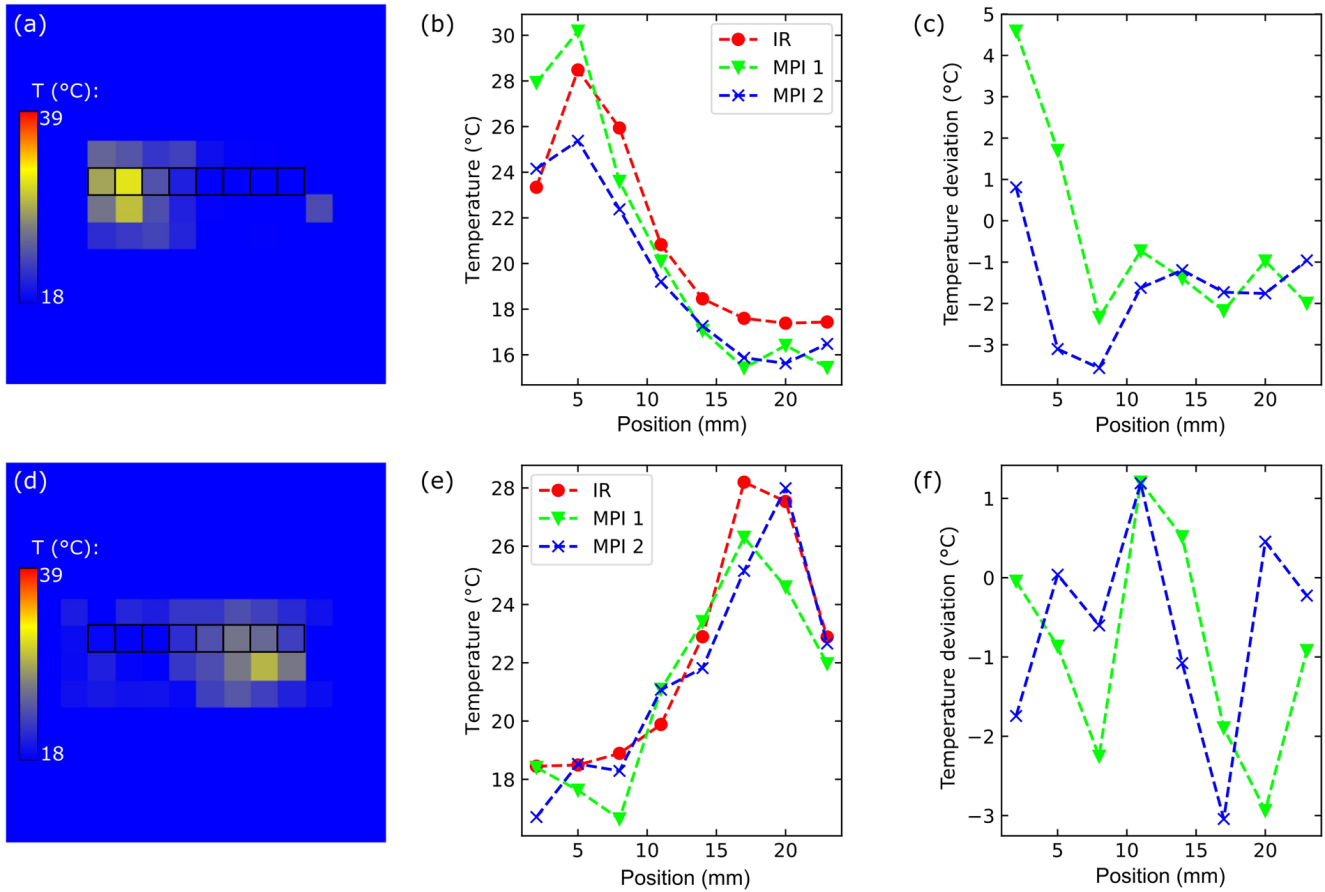
Comparison of MPI and IR temperature image results of laterally heated L93 phantom at *t* = 151 s: (**a**) MPI temperature image heated at the left side, (**b**) comparison of rectangle-marked elements of the MPI temperature image with IR camera reference and (**c**) corresponding temperature deviations. (**d–f**) Phantom is heated at the right side.

A test of the presented temperature MPI approach to locate heating throughout the whole phantom and FoV can be found in Fig. 8 illustrating a comparison similar to Fig. 7, but with a copper wire stuck laterally into the phantom. The temperature hot spot – either on the right or left side – is located correctly in the MPI temperature images. A quantitative comparison of line profiles – taken from the IR camera image and two layers of the MPI temperature image stack – shows an acceptable agreement between MPI and IR results. In this case, the temperature deviations of the line profiles amount to maximal 4.5 °C, again for temperatures located at the edges of the phantom. This experiment indicates - in agreement with our initial investigations – that the temperature coefficient *m* that we determined in the center has no strong spatial dependence.

**Figure 9:**
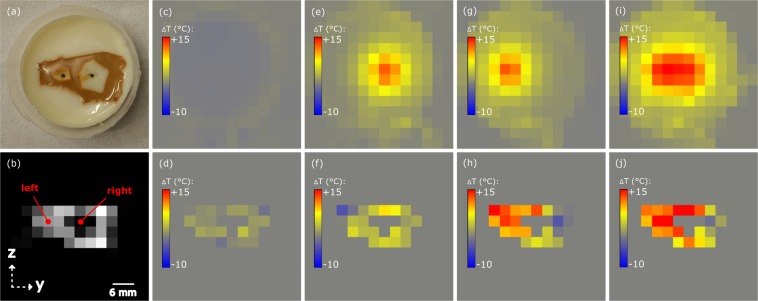
MPI temperature imaging of liver tumor ablation phantom: (**a**) Photography of L93 phantom with two differently sized tumors (SPION-free white spots) and (**b**) corresponding layer of reconstructed MPI data indicating copper wire position. IR (top row) and MPI temperature images (bottom row) illustrate temperature changes (**c,d**) without heating any tumor and heating tumor at (**e,f**) right, (**g,h**) left and (**i,j**) both positions (*t* = 172 s). The reconstructed images present the scanner’s x-y-plane.

The principle feasibility of MPI to monitor the temperature of healthy liver tissue surrounding tumors during thermal ablation therapies was evaluated with the phantom shown in Fig. 9a. The size of the denatured protein structure with embedded L93 (c(Fe) = 12.8 mM) is comparable to the previous phantoms. Inside the structure two SPION-free protein areas (white spots) can be found, representing two liver tumors with different sizes. At least the larger SPION-free liver tumor caused a clearly visible gap in the corresponding layer of the reconstructed MPI image, as demonstrated in Fig. 9b. This reflects MPI images recorded in small animal investigations^10,20^. A MPI scan without heating any tumor showed slight temperature changes in MPI and IR reference images (Fig. 9c,d). A separate and parallel heating of the tumors resulted in expected positive temperature changes around the tumors of up to 15 °C, which are in qualitative agreement with IR reference images (Fig. 9e–j). However, the exact temperature distribution of the MPI images differs from the expected results; for instance, some voxels show a significantly reduced temperature rise or even cooling.

## Discussion

The relative MPI signals of all investigated SPIONs showed an approximately linear relationship to temperature in the performed MPI scans, stated as positively temperature-dependent. This is partly contrary to previous investigations, for instance^34^, found a positive dependence for perimag and a negative one for ferucarbotran (Resovist). However, only one signal frequency component of liquid SPION suspensions was analyzed hampering a direct comparison of results, especially, when considering the frequency dependence of the temperature influence in the MPS results (Fig. 3). In general, the exact SPION structure and environment have a strong impact on the temperature dependence in MPI applications. In our case, the SPION immobilization with denatured protein blockades a free SPION rotation (Brownian relaxation process). Thus, the observed positive temperature dependence is dominated by the temperature dependence of the internal rotation of the SPIONs magnetization due to alternating magnetic fields (Néel relaxation process). This is in agreement with temperature-dependent MPS measurements comparing mobile (Brown) and immobile (Néel) SPION samples^28^. The relationship between SPIONs static magnetization and temperature is theoretically characterized by a negative dependence^22^ and, consequently, less relevant in our case.

The measured MPI temperature images agreed well with IR camera results. Induced hot spots were correctly located throughout the phantom and a mean absolute deviation of 1 °C was found in the performed comparisons shown in Figs. 7,8. Since IR cameras measure surface temperatures, MPI results from two different layers are shown. Here, no significant differences with respect to deviations between MPI and IR results could be seen. This indicates that a possible depth dependence of temperature is at least for these comparisons negligible.

The investigated temperature MPI approach benefits from a reduced calibration effort. An exact and constant sample temperature regulation for system matrix acquisition at different temperatures was not required enabling easier and quicker calibrations. However, most notably in the boundary areas of the particle distribution, MPI temperature images yielded less accuracy and even measured negative temperature changes. This apparent phantom cooling is physically not plausible, could not be identified with IR camera measurements and cannot be explained by the slight spatial dependence of *m*. In fact, it represents the main limitation of this MPI temperature imaging approach based on only one system matrix. We see two reasons for this. First of all, the regularized image reconstruction leads to non-local changes in the calculated particle concentration. In particular, if there is mismatch between the state of the particles in the system matrix and the actual measurements, we expect a non-zero residual. During image reconstruction the residual is minimized, which can lead to non-local artifacts. Consequently, a mismatch in the temperature in a specific location might lead to an artifact in another location. This issue will be partly addressed by the regularization but non-local artifacts will still appear for high temperature changes. Second, these negative temperature estimations primarily appear at the boundaries of the particle distribution where the MPI signal is already low. Consequently, these values are prone to a larger relative error which could increase the cooling. One way to handle the non-zero residual issue could be the usage of multi-color temperature MPI, which would use two or more system matrices at different temperatures and in turn allows integrating the temperature estimation directly into the reconstruction process in contrast to the pixel-wise post-processing strategy that we applied in this work.

In the case, that an optimized reconstruction strategy would solve the previously described limitation and to highlight the principle potential of magnetic nanoparticles as temperature probes in imaging, an uncertainty estimation is presented: Assuming MPI temperature monitoring of SPION-loaded tissue heated from a body temperature of 37 °C to 50 °C, following single uncertainties are considered for an exemplary estimation of the overall uncertainty of ***T***_**MPI,t**_ according to Eq. (2). The uncertainty of *m* from the temperature coefficient analysis is 0.00294 K^-1^. The uncertainties of the MPI images recorded at 37 °C and 50 °C, required for the calculation of ***MPI***_**rel,t**_, are estimated at 0.4% of the MPI particle concentration of the corresponding image element. These values are based on the relative standard deviation of one central MPI image element taken from a 40 times repeated MPI scan of the L93 phantom from Fig. 6 without stuck copper wire. The uncertainty of the initial tissue temperature distribution ***T***_**MPI,0**_ is expected to be around 1 °C, when assessing the core body temperature with a medical thermometer. Finally, propagation of error – applying linearization of Eq. (2) with first-order Taylor series expansion – yields a maximum uncertainty of 5 °C for a central MPI temperature image element. This result represents a fair accuracy for absolute temperature images, for instance, IR camera Testo 885 possesses an accuracy of ±2 °C. The sensitivity of MPI temperature images – characterizing the method’s capability to detect temperature changes – amounts to 0.8 °C for the described example. This result coincides with the signal variation identified in Fig. 6c.

The MPI voxel size of 3 × 3 × 3 mm^3^ represents a limitation for a murine liver, however, for a human thermal ablation therapy an adequate resolution. In fact, the limited FoV has to be enlarged for human applications. Upscaling MPI for human dimensions is possible in principle^35,36^ but a human sized scanner cannot be expected in the near future. Fortunately, the thermal response time of SPION to local temperature changes is faster than the utilized MPI scanner’s repetition time of approximately 21.54 ms and much faster than heat transfer in tissue^37^. Assuming an upscaled version of our scanner, half the gradient strength would still be sufficient for a 3 × 3 × 3 mm³ voxel size. Using the focus-field approach^38^, a scan of a 0.25 L volume per second with our preclinical system would be possible, still meeting clinical needs. Phantom heating induced by eddy currents seems not appropriate for clinical applications, since heating control is directly linked to temperature image acquisition possibly causing inadvertent tissue damage. In addition, tumor temperatures of even more than 100 °C can be found in thermal ablation therapies, which could hardly be realized with the copper wire approach. Thus, laser induced heating with optical fibers represents a proper solution. The application of radiofrequency or microwave ablation devices is likely hampered by electromagnetic interferences caused by the MPI field.

## Conclusion

The potential of MPI to monitor the temporal and spatial temperature distribution during thermal ablation of liver tumors was analyzed. The prepared phantoms based on denatured protein with embedded SPION were heated up to 70 °C yielding relative linear and positive temperature dependencies. For the optimal SPION, L93, a linear model was constructed to calculate MPI temperature images. Comparisons with simultaneously recorded IR camera measurements present good quantitative agreements with a mean absolute deviation of 1 °C. However, invalid phantom cooling is partly found most notably in the boundary areas of the particle distribution. This effect is most likely explained by non-local reconstruction artifacts and represents a limitation of the practical realization of this MPI temperature imaging approach. Nevertheless, the principle feasibility of MPI-guided liver tumor ablation could be demonstrated and the identified SPION temperature sensitivity resulted in acceptable uncertainties.

## Data Availability

Most of the data generated or analyzed during this study are included in this published article, all source datasets generated during and/or analyzed during the current study are available from the corresponding author on reasonable request.
